# Abrupt conclusion of the late Miocene-early Pliocene biogenic bloom at 4.6-4.4 Ma

**DOI:** 10.1038/s41467-021-27784-6

**Published:** 2022-01-17

**Authors:** B. – Th. Karatsolis, B. C. Lougheed, D. De Vleeschouwer, J. Henderiks

**Affiliations:** 1 Department of Earth Sciences, Uppsala University, Villavägen 16, 75236 Uppsala, Sweden; 2 MARUM-Center for Marine and Environmental Sciences and Department of Geosciences, University of Bremen, Leobenerstr 8, 28359 Bremen, Germany; 3 Institute of Geology and Palaeontology, Westfälische Wilhelms-Universität, University of Münster, Corrensstr 24, 48149 Münster, Germany

**Keywords:** Palaeoceanography, Palaeoclimate

## Abstract

The late Miocene-early Pliocene biogenic bloom was an extended time interval characterised by elevated ocean export productivity at numerous locations. As primary productivity is nutrient-limited at low-to-mid latitudes, this bloom has been attributed to an increase or a redistribution of available nutrients, potentially involving ocean-gateway or monsoon-related mechanisms. While the exact causal feedbacks remain debated, there is even less consensus on what caused the end of the biogenic bloom. Here, we compile Mio-Pliocene paleoproductivity proxy data from all major ocean basins to evaluate the timing and pacing of this termination. This systematic analysis reveals an abrupt and sustained reduction in low-latitude ocean productivity at 4.6–4.4 Ma. The decline in productivity coincided with a prolonged period of low orbital eccentricity and a shift towards lower-amplitude obliquity, an astronomical configuration linked to reduced East Asian Monsoon intensity and decreased riverine nutrient supply.

## Abstract (web summary)

What caused the end of the high ocean productivity in the tropics in late Miocene-early Pliocene is debated. Here, deep-sea sediment records reveal that productivity abruptly declined 4.6 million years ago, potentially linked to reduced seasonality and monsoon intensity that led to decreased nutrient supply to the oceans.

## Introduction

Ocean productivity is driven by marine primary producers (e.g. diatoms and coccolithophores) that rely on the availability of nutrients, which are quickly exhausted in the sun-lit surface waters if not replenished. Changes in primary productivity are reflected in the amount of matter that is exported to the deep ocean (export production) which is partly buried into the sedimentary archive. Although only a small fraction of surface water production is recorded in marine sediments, on geological time scales, changes in the accumulation of biogenic sediments are linked to past changes in ocean productivity. As a prime example, the late Miocene to early Pliocene biogenic bloom^1^ has been described from various locations in the Pacific, Atlantic and Indian Ocean and approximately spans from 9 to 3.5 Ma^1–5^. It is characterized by regionally elevated rates of biogenic silica and carbonate accumulation^2,4,6,7^, an expansion of oxygen minimum zones (OMZs) in the Indian Ocean^8^, as well as increased burial of phosphorus (P) and barium (Ba) in all major oceans^9,10^. Combined, these observations mirror significant increases in marine paleoproductivity (PP) during this time. The biogenic bloom may have been diachronous at different locations and various mechanisms have been proposed to explain its development. On a global scale, hypotheses range from increased nutrient input into the ocean^10^ and intensification of the late Miocene Asian monsoon^11^, to significant reorganizations in ocean circulation^12^ and shifts in depocenters^13,14^, as well as intensification of regional upwelling^1,2^.

Although the existing records of increased marine PP have been studied in detail, there is still limited knowledge regarding the possible climatic drivers/feedbacks during the biogenic bloom and, especially, the reasons why it terminated in the early Pliocene. Studies disagree on the exact timing of maximum PP, both within and between different ocean basins, while little attention has been given to potential synchronicity of events towards the end of the biogenic bloom.

The main period of increased PP is estimated to have occurred approximately between 7 and 5 Ma, with a termination between 4.6 and 3.5 Ma^2,5,7,15^. In the California Current region, however, the main decrease in diatom productivity occurred considerably earlier (7.5 Ma^16^) than elsewhere and this difference in timing has been related to a shift in biogenic silica depocenters resulting from the closure of the Central American Seaway (CAS)^1^. Tectonic reorganization has been repeatedly proposed as the main trigger for ocean circulation changes that led to the observed differential patterns of increased PP across basins during the early Pliocene^6,17^. However, while tectonic plate movements are a slow process, the observed productivity changes are often abrupt and sometimes lacking clear geographical patterns controlled by ocean gateways^7^. This time-scale discrepancy between slowly-paced tectonic reconfigurations (million-year processes) and more abrupt productivity changes (100–200 kyr processes) raises the need for the evaluation of other potential mechanisms behind oceanwide changes in productivity.

**Fig. 1:**
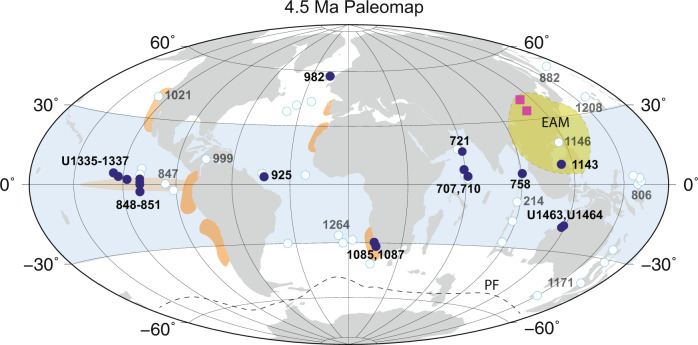
Early Pliocene (4.5 Ma) paleogeographic reconstruction showing deep-sea drill sites (ODP and IODP) discussed in this study. In total, 25 paleoproductivity records from 18 (mostly low-latitude) sites were compiled (closed symbols). Other deep-sea sites (open symbols) and terrestrial records from Central China (squares) were evaluated but not included in the final data compilations. Modern (perennial and equatorial) upwelling regions are indicated as orange shadings. EAM: East Asian Monsoon (green shaded area). PF: Polar Front (Southern Ocean; dashed line). Map generated on www.odsn.de (webpage consulted in 2020).

Here, we present a compilation of globally distributed PP proxy records, mainly from low latitudes (30°N–30°S; Fig. 1), including biogenic silica and carbonate mass accumulation rates (BSMAR, CMAR), as well as benthic foraminifera and nannofossil (coccolith) accumulation rates (BFAR, NAR), to trace the final stages of the biogenic bloom. The compilation is based on a robust re-evaluation of available records and reveals a synchronous PP decline in low-latitudes, constrained to within 200,000 years during the early Pliocene. A number of possible causal mechanisms could explain these observations, including a potential link to Earth’s orbital configuration.

## Results and discussion

### Data compilation

**Fig. 2:**
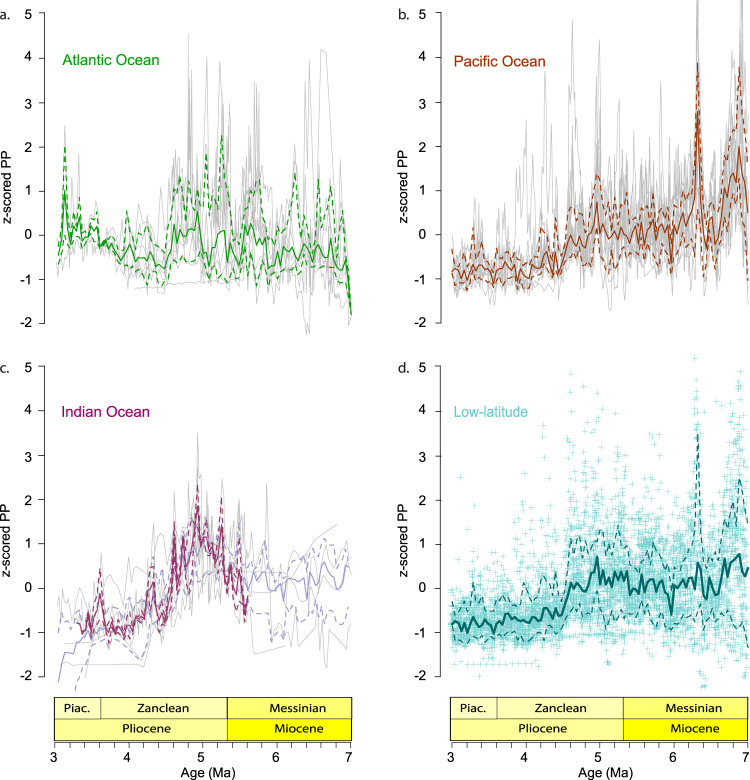
Standardized marine paleoproductivity (PP) records across the late Miocene - Pliocene. The median (solid lines) of binned and z-scored PP data (grey lines in **a**–**c** and crosses in **d**) is shown for **a** the Atlantic Ocean, **b** the Pacific Ocean, **c** the Indian Ocean and **d** all vetted records combined. Dashed lines indicate the 15.9 and 84.1 percentiles (i.e. the central 68.2 percentiles) of the binned data. The Indian Ocean compilations (**c**) include the median of input records that scored 4 or 5 only (dark purple line) and that calculated including records that scored 3 (light purple line). Piac. = Piacenzian. Source data for this figure are provided as a Source Data file.

The raw PP proxy data from the different oceans are complex and non-uniform, meaning that it is difficult to draw comparisons (Supplementary Figs. 1–5). To alleviate this issue, we first vetted proxy records based on an objective scoring system that takes into account data resolution and the quality of the available geochronology (see Methods for details on the 1 (bad) to 5 (excellent) scoring system). Second, we standardized all proxy records, by expressing productivity data as z-scores. The vetted and standardized data compilation reveals that the late Miocene to early Pliocene was a time of increased ocean productivity at the majority of the studied records (Fig. 2). During the early Pliocene, a marked reduction in PP is observed in most of the records and in all oceanic basins. The only site to record a significant increase in BSMAR at that time is the eastern equatorial Pacific (EEP) IODP Site U1337, although this site has evidence for significant sediment focusing and is, therefore, considered to not accurately reflect original deposition^7,16^.

The compilation also reveals significant variability in PP prior to the early Pliocene decrease. The Atlantic Ocean shows the highest median PP state during ~6–4.5 Ma (Fig. 2a), while this is the case from ~7 Ma to ~6 Ma in the Pacific (Fig. 2b) and ~5.5–4.2 Ma in the Indian Ocean (Fig. 2c). Despite these differences in PP which occur mainly during the late Miocene, a signal of significant reduction is evident in all three major oceans during the early Pliocene (~4.6–4.2 Ma; Fig. 2a–c). This reduction becomes even more abrupt (~4.6–4.4 Ma), if we exclude Indian Ocean records that scored 3 (no such records in the Pacific and Atlantic), retaining only the data from the NW Australian shelf (scores 4 and 5) (Fig. 2c). PP in the Pacific and Indian Ocean did not return to higher values for the rest of the Pliocene, whereas a period of higher PP is suggested to have occurred in the Atlantic after 3.5 Ma. However, this (apparent) increase is driven by a single record (tropical ODP Site 925, CMAR), and in absence of data coverage between 3.5–3 Ma for most of the selected Atlantic Ocean records in our analysis, it may not be representative for the Atlantic basin as a whole.

When all PP records from all oceans are combined in a single compilation, a downward step in the median PP state is evident in the early Pliocene (Fig. 2d). Specifically, late Miocene to earliest Pliocene differences between oceans are smoothed and the synchronous reduction in PP (4.6–4.4 Ma) becomes more prominent. The values remain low after this transition and for most of the remaining Pliocene, pointing to a sustained reduction in a range of PP proxies. Our data compilation contains a strong signal from equatorial and perennial upwelling areas (such as the EEP and the Benguela upwelling areas) and mainly includes sites from low latitudes (blue shading in Fig. 1). Nevertheless, many other contributing sites are situated outside upwelling areas. For example, sites located on the NW Australian shelf (IODP Sites 1463, 1464), in the North Atlantic (ODP Site 982), the South China Sea (SCS) (ODP Site 1143) and the tropical Western Atlantic (ODP Site 925) also record the PP decline during the early Pliocene (Supplementary Figs. 2–4). This supports that, at least with the vetted sites examined in this study, the reduction in productivity was a widespread event characteristic of the low latitudes and not restricted to upwelling regions.

### Early Pliocene ocean productivity and the end of the biogenic bloom

The observed Early Pliocene stepwise decrease in ocean productivity occurred rapidly (~200 kyrs, between 4.6–4.4 Ma) and globally (at low latitudes in all ocean basins; Fig. 2). Productivity remained on an average lower level since. This change could be interpreted either as a sharp demarcation indicating a sudden end of the biogenic bloom, or as an important step towards its final stages, and may involve several paleoceanographic and paleoclimatic mechanisms to explain its possible causes.

The first mechanism includes the role of low-latitude divergence zones, mainly located in the northernmost Indian Ocean and the Arabian Sea (seasonal upwelling), the EEP (equatorial upwelling) and the Benguela (perennial upwelling). Intensified upwelling in the first two systems has been previously proposed as a driver for elevated productivity^7,18^, whereas changes in the Benguela system were mainly attributed to ocean-wide increase in nutrient supply and not to a regional upwelling intensification^14^. In reverse, a weakening of upwelling intensity could have led to a reduction in productivity and there is, for example, evidence for weakening of the EEP upwelling after 4.5 Ma^19^. However, if upwelling was the sole driver of change, we would expect to see a geographically restricted response within or near upwelling regions. Instead, we observe that the synchronous PP reduction between 4.6 and 4.4 Ma is not restricted to upwelling regions only. Western Pacific records from the Ontong Java Plateau (ODP Sites 803, 804, 806, 807) also record a phase of CMAR reduction at ~5 Ma^18^, although their age models did not make the threshold of our scoring system to be included in the compilation (Supplementary Table 2). In higher-resolution BSMAR records from the SCS (ODP Site 1143), the PP decrease is evident between 4.6–4.4 Ma. The event is also strongly expressed elsewhere (North Atlantic, NW Australian shelf), strengthening the hypothesis of significant changes in the nutrient inventory.

The second mechanism invokes shifts in the main depocenters and biologic productivity compensation elsewhere, driven by changes in ocean circulation and tectonic reorganizations. Early biogenic bloom studies suggested that an increase in nutrients in high-productivity regions occurred at the expense of low productivity areas^13^, or that nutrient levels increased in the Pacific at the expense of those in the Atlantic Ocean^13,15^. Hermoyian and Owen (2001)^10^ challenged this hypothesis, proposing that the biogenic bloom also occurred in low productivity areas of the Atlantic and Indian Ocean, although the latter records failed our age model criteria and therefore this interpretation should be considered with caution. Despite the critique of Hermoyian and Owen (2001)^10^, the depocenter-shift hypothesis still has merit to explain the sustained PP reduction in low latitudes after 4.4 Ma, as it is supported by the indisputable observation that the history of biogenic sedimentation, even within basins, is extremely complex. The early Pliocene increase in BSMAR at North Pacific ODP Site 882^20^, as well as the increases in CMAR at ODP Site 1021 (located at the California Margin)^21^ and at ODP Site 1208^18^ could indicate a shift of the main locus of biogenic deposition away from the tropics and towards the North Pacific since 4.6 Ma. At that time, there is evidence for an intensification of the Gulf Stream and increased deep-water formation in the North Atlantic, related to a major shoaling phase of the CAS^22^, which would have led to an invigoration of thermohaline circulation and an intensification of the North Pacific upwelling^20^. Stronger SST gradients between the equatorial Pacific and the California Margin driven by cooling in the latter region^23^ (ΔSST meridional; Supplementary Fig. 8), could be linked to the intensification of the North Pacific gyre, which brings cold water in the area through the California Current and intensifies regional upwelling^24^. This chain of events adds a significant ocean circulation component to the mechanisms controlling the termination of the biogenic bloom in low latitudes through a PP compensation in higher latitudes. In the Southern Hemisphere, the Southern Ocean is the main candidate for a similar high-latitude PP shift in depocenters. However, a compilation of diatom records from 34 deep-sea sites does not show any significant trend during the early Pliocene nor any sustained changes in BSMAR after 4.6–4.4 Ma^25^, although a southward movement of the Polar Front (PF) and significant turnover rates in diatom species, possibly linked to orbitally-paced cooling events, have been reported for this time interval^25,26^. To further demonstrate the complexity of PP patterns, differential biogenic sedimentation has in some cases been reported even within small distances in the same basin. For example, ODP Site 1085 shows a rebound in CMAR after 3 Ma while the biogenic bloom seems to have persisted at Site 1264 until 3 Ma^27^.

While ocean circulation changes due to gateway restrictions cannot be excluded, the documented low-latitude PP decrease would be hard to explain solely by tectonic movements, since it demonstrates synchronicity across basins and only lasted ~200 kyr. The CAS shoaling is the only major mid-latitude tectonic reorganization to have occurred during this time and operates on much longer timescales, with major activation stages at 4.2 Ma^28^ and after 3.2 Ma^29^. The Indonesian Throughflow (ITF) restriction occurred much later during the Pliocene^30,31^. It is therefore worthwhile to investigate other, shorter-term mechanisms that could have combined their effects with the slower tectonic movements to generate the patterns observed. This brings us to a third mechanism that has been associated with the biogenic bloom: Changes in the total nutrient inventory of the oceans, as well as redistribution of these nutrients within the low latitudes. Filippelli (1997)^11^ hypothesized that the biogenic bloom was linked to intensified monsoon and thus increased nutrient supply to the oceans. Although this hypothesis is unlikely to represent the sole driver of the biogenic bloom and has been challenged by the relatively low uplift rates of the Himalayas after 10 Ma^32^, a link between monsoonal intensity and low-latitude nutrient availability via river runoff is a feasible factor that could have enforced broad-scale changes in PP on shorter, climatic timescales. The main candidate influencing nutrient supply in the low-latitude oceans is the East Asian Monsoon (EAM). Being the largest monsoonal system, it controls precipitation patterns over a vast area in mainland Asia (Fig. 1). The major Asian rivers, that are directly influenced by changes in EAM precipitation, have been estimated to account for up to 40–50% of the global inorganic and organic C fluxes^33–35^ and more than one-third of the global riverine dissolved Ca flux from the land to the ocean^36–38^. Combined with its wide latitudinal reach, EAM activity can significantly affect ocean productivity, either through the input of biologically important elements into the ocean or through the intensification of seasonal winds and upwelling^39,40^. A variety of terrestrial records exist for the early Pliocene, indicating a very complex history of the EAM over Mainland China. All possible scenarios have been proposed in recent studies, including stages of intensification^41–43^, weakening^44^, and migration of the EAM^45^; but most agree on a weakening or a northward shift of the EAM rain belt at ~4.3–4.2 Ma^45–47^ (Supplementary Fig. 7). The direct runoff evidence from SCS sediment records, that are the most relevant when examining the link between terrestrial processes and river outflow, shows a long-term weakening of the East Asian Summer Monsoon (EASM) since the Miocene^44^. More specifically, a reduction in chemical weathering, inferred from the elemental composition and clay mineral content of the sediments, occurs in good accordance with our PP decrease phase^44^ (ODP Site 1146 records in Supplementary Fig. 7b), strengthening the hypothesis that weathering and nutrient runoff through major riverine systems weakened significantly during the early Pliocene. It is important to note, however, that most of the available monsoon records, including those from SCS, have age models that would score within the lower end of our grading system (scores 2–3; Supplementary Table 3). Direct comparisons of our PP compilation with these records should therefore be done with caution. Further investigation, including EAM proxy compilations according to age model fidelity and terrestrial-marine record correlations, will be needed to validate the possible effect of monsoonal intensity on low-latitude PP.

### Potential links to astronomical parameters and early Pliocene paleoclimate

**Fig. 3:**
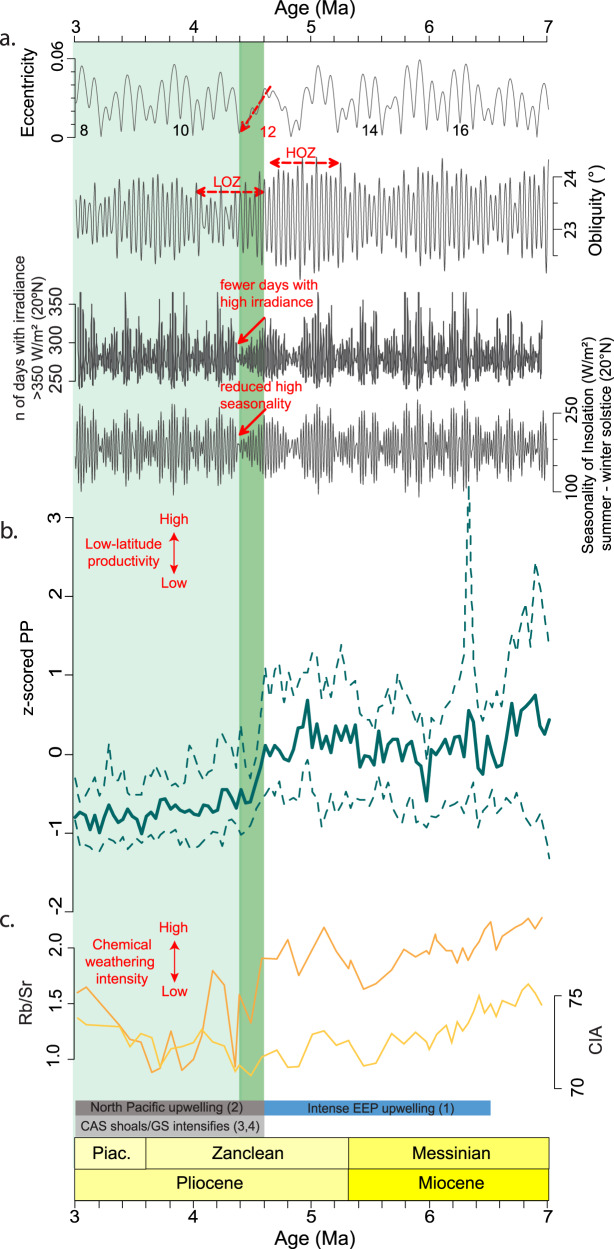
Synthesis of orbital configuration, marine PP and chemical weathering intensity for the late Miocene to early Pliocene. **a** From top to bottom: Laskar 2011^53^ eccentricity solution; Laskar 2004^74^ obliquity solution. HOZ = High Obliquity Zanclean and LOZ = Low Obliquity Zanclean; Number of days with irradiance ≥ 350 W/m^2^ at 20 °N; difference between summer and winter solstice irradiance (seasonality of insolation) at 20 °N. **b** Median standardized marine PP compilation, dashed lines indicate 15.9 and 84.1 percentiles (i.e. the central 68.2 percentiles) of the binned data (see Fig. 2d). **c** South China Sea records of the Rb/Sr ratio and the CIA index (100 × Al_2_O_3_/Al_2_O_3_ + CaO + Na_2_O + K_2_O) at ODP Site 1146. Both proxies are used to describe variations in EASM and chemical weathering intensity^44^. Coloured horizontal bars indicate: (1) Period of intensified EEP upwelling^19^, (2) Period of North Pacific upwelling intensification^20^. (3,4) Main phases of Central American Seaway (CAS) shoaling and Gulf Stream (GS) intensification^22,29^. Vertical green shadings highlight the early Pliocene PP decline (4.6–4.4 Ma) and sustained lower PP in its aftermath as identified in this study. Source data for this figure are provided as a Source Data file.

The short duration of the observed step-wise PP decrease (~200 kyr) brings early Pliocene astronomical forcing into focus. Astronomical forcing has not yet been investigated in detail in the context of the biogenic bloom termination, yet it could be of importance for the observed productivity decrease through the modulation of monsoonal intensity. A wide range of evidence from empirical proxy records and numerical simulations indicate that the EAM is highly sensitive to variations in insolation forcing, whereby high summer insolation regimes typically lead to increased precipitation, weathering and river runoff and vice versa^48–52^. The eccentricity solution^53^ holds a so-called eccentricity node close to the interval of PP decrease (4.8–4.4 Ma; Fig. 3a). This orbital configuration marks the termination of a 2.4 Myr eccentricity cycle. The decline in PP between 4.6 and 4.4 Ma coincides with the period of consistently low eccentricity, which leads to subdued precession-controlled insolation extremes at low latitudes. Consequently, the number of days at 20°N with peak irradiance is systematically reduced (Fig. 3a; Supplementary Fig. 6), which in turn could have reduced monsoon intensity and therefore chemical weathering and river runoff. During the same interval, obliquity switches to a lower-amplitude regime as well (from the High Obliquity Zanclean HOZ to the Low Obliquity Zanclean LOZ; Fig. 3a). Evidence from GCMs indicate that obliquity plays a secondary, yet significant, role in controlling precipitation patterns over Southeast Asia. Obliquity maxima generally lead to wetter conditions compared to minima^54,55^. The fact that obliquity is reduced in amplitude and, therefore, obliquity maxima did not exceed 23.9° during the entire interval of PP reduction, could have enhanced the effect of eccentricity in muffling precipitation extremes and river runoff. This combination of eccentricity and obliquity is rare in geologic history and unique for the biogenic bloom interval. It could have facilitated the establishment of a climate state with reduced seasonality extremes, with weaker monsoonal dynamics and chemical weathering, and with a nutrient flux that was insufficient to sustain low-latitude marine PP. The effect of nutrient limitation could have been further amplified in a warm, stratified ocean with very low zonal gradients (ΔSST zonal; Supplementary Fig. 8) that characterised low latitudes during this time^56,57^. At the same time, proxy records reveal an increase in meridional temperature gradients^23^ (Supplementary Fig. 8), with climate models indicating that this could have led to a further increase in tropical ocean stratification^58,59^. Such steepening of meridional gradients could have also been influenced by the early Pliocene orbital configuration, and specifically the obliquity of Earth’s rotational axis, since it controls the latitudinal distribution of insolation^60^. A prolonged period without high obliquity, like for example the LOZ, would have resulted in an increase in the latitudinal insolation gradient, which in turn could have contributed to the steepening of meridional temperature gradients^61,62^. To summarize, a combination of reduced monsoonal dynamics and nutrient input in tropical latitudes driven by the orbital configuration, strong ocean stratification and an increase in meridional gradients, could have led to the marked decrease in low-latitude PP within ~200 kyrs.

### Fossil plankton evidence

The rather abrupt and sustained reduction in PP points to drastic ecological (and possibly evolutionary) changes within the early Pliocene plankton assemblages. Neogene patterns in CMAR have been shown to be primarily driven by calcareous nannoplankton (mainly coccolithophores) export production (and sub-lysocline dissolution)^18,63^. Coccolithophores constitute the bulk of primary productivity in low-latitude, stratified waters where species diversity is also highest. Although micropaleontological details are lacking for most records in our compilation, it is important to highlight that the decrease in NAR over the NW Australian shelf (IODP Sites U1463-1464) is paired with a major shift in species dominance in the fossil coccolith assemblages^64^. We can only speculate on the physiological differences and ecological tolerances of ancient (and now extinct) marine algae, but cell geometry does correlate broadly with cellular growth rates (or exponential vs. stationary growth phases) in modern bloom-forming taxa^65,66^. Preliminary data at IODP Site U1464 (Supplementary Fig. 9) reveal that the *Reticulofenestra* species that dominated late Miocene-early Pliocene assemblages had smaller cells and much higher burial fluxes (NAR) than their successors, small *Gephyrocapsa* species that became dominant at ~4.42 Ma^64^. As for ecological tolerances, small *Gephyrocapsa* may have coped better with warm, stratified conditions than small *Reticulofenestra*, which is often labelled as an opportunistic bloom-forming taxon with a wider tolerance for highly variable conditions (Auer et al., 2019^67^ and references therein). The shift from small *Reticulofenestra* to *Gephyrocapsa* is not unique to the NW Australian shelf records^64^. It has been reported as the (base of) small *Gephyrocapsa* acme in a range of biostratigraphic studies in e.g. the Mediterranean^68^, equatorial Atlantic^69^, Caribbean and EEP^70,71^; yet, the base of this dominance interval appears to be diachronous between ~4.4 Ma (base biozone CN11a) and ~3.8 Ma (top biozone NN15). Its correlation (and relationship) to changes in NAR or other PP proxies need further scrutiny in future high-resolution analyses alongside a rigorous age model review of existing records. Together with high-latitude phytoplankton reorganisations such as the diatom species overturn reported for the Southern Ocean^25,26^, fossil plankton evidence will likely offer some valuable additional information about major changes in global ocean productivity following the end of the biogenic bloom.

Although many questions still remain, such as whether productivity was decreased overall in the ocean or increased in equal proportions towards higher latitudes, our data compilation offers compelling evidence for a significant and synchronous decline in ocean productivity in low latitudes of all ocean basins between 4.6–4.4 Ma. The close inspection of the individual geochronologies of these paleorecords proved crucial to put them on a common timeline, to resolve patterns between or within ocean basins and to discuss potential mechanisms leading to the biogenic bloom termination. The most plausible scenario seems to include a combined effect of reduced nutrient availability in low latitudes and a shift in depocenters favouring biogenic sedimentation in the North Pacific. The conspicuous correlation with a rather unique orbital configuration, representing a 200-kyr period of anomalous climate stability (lacking extremes in low-latitude precipitation as simulated in GCMs with fixed orbital configurations), may point to a prolonged situation where continental runoff and nutrient availability in the photic zone proved insufficient to sustain the biogenic bloom. Until time-varying, coupled GCM-biogeochemical models can realistically simulate the tentative relationships between ocean productivity, the East Asian Monsoon and orbital configurations, we suggest that high-resolution fossil plankton assemblage studies may be one of the most direct ways to further detail the regional and global ecological implications of this early Pliocene event.

## Methods

### Compilation of paleoproductivity records

A total of 58 deep-sea records from 51 International Ocean Discovery Program (IODP) and Ocean Drilling Project (ODP) sites were considered (Fig. 1; Supplementary Tables 1 and 2). From these, 25 records from 18 sites were selected and compiled in the final analyses. The selected records include (mass) accumulation rates (in g/cm^2^/kyr or N/cm^2^/kyr) of biogenic silica (BSMAR), CaCO_3_ (CMAR), benthic foraminifera (BFAR) and calcareous nannofossils (NAR). Sites are distributed across multiple basins at mainly middle to tropical latitudes of three major oceans. All records cover the late Miocene and most of the Pliocene (7–3 Ma) and span the past four decades of research, containing data with various temporal resolution and different age-depth model approaches (Supplementary Table 1). No specific latitudes or records were a priori selected in the analyses.

To evaluate each record, we applied a criteria-based scoring system (1–5 [bad-excellent]) that takes into consideration the sample spacing of each proxy measurement as well as the methodology and temporal resolution of the geochronology (Table 1). Only records with scores of 3 or higher (25 records) were included in our final compilations, to ensure age model robustness and comparability across individual records and ocean basins. Details on the resolution and the citations of all considered records are listed in Supplementary Tables 1 and 2.

**Table 1:** Description of the 1–5 scoring system for paleoproductivity records evaluated in this study.

Score	Description
1	Major sample gaps close to the period of interest (end of the biogenic bloom)
2	Low-resolution biostratigraphy and chronostratigraphy (<1 age-depth tie point per 1 Myr between 3–5 Ma, biostratigraphic and magnetostratigraphic zone estimation from core catcher samples or low sampling resolution)
3	High-resolution biostratigraphy and chronostratigraphy (>1 age-depth tie point per 1 Myr between 3–5 Ma)
4	Astronomical tuning and ≤50 samples between 4–5 Ma
5	Astronomical tuning and >50 samples between 4–5 Ma

In several cases (Indian Ocean ODP Sites 707, 710, 721 and 758) CMAR data was recalculated, using the combination of biostratigraphic and magnetostratigraphic tie points summarised in Dickens and Owen (1999)^2^, but with updated nannofossil datum ages^72,73^.

Each individual record was trimmed to 7–3 Ma, so that all records cover the same time interval, and subsequently standardized by means of a z-score calculation (subtracting the mean value of the record from each data point before dividing by the record’s standard deviation; Supplementary Material). Z-scored individual records were then compiled for each ocean basin before all selected records were combined (Supplementary Figs. 1–4). Thereafter, the z-scored data was grouped into 40 kyr time bins. The chosen time bin falls close to the median temporal resolution (samples/Ma) of all the records we used (Supplementary Table 1). Other time bins were tested, ranging from 20 to 100 kyr, with no significant effect on the main findings (Supplementary Fig. 5). The median and 68% percentiles (asymmetrical due to non-normally distributed data) of the z-scores in each time bin were subsequently calculated.

Finally, to avoid the bias of a strong signal derived from one ocean basin with multiple records skewing our final compilation (e.g. Pacific Ocean EEP records), we made distinct inter-ocean observations before each compilation step. This way we made sure that potential signals that stand out as being synchronous and uniform were recorded in all oceanic basins before combining all in the final compilation.

### Potential sampling biases and age model limitations

We took into consideration all available PP records from major (including review and compilation) studies across the time interval of interest (Fig. 1; Supplementary Table 1, Supplementary Table 2). Our age model criteria drove the final selection for our compilations, as opposed to a latitudinal, site or proxy-specific criteria. However, most of the final compiled records are biased towards low latitudes (Fig. 1). There is a clear lack of high-resolution records (the ones that would score 35; Table 1) spanning the late Miocene—early Pliocene from higher latitudes (>30°) in either hemisphere.

The Atlantic is the only ocean with mid-latitude, high-resolution CMAR records (ODP Site 982)^4,5^. Since we did not a priori exclude any sites or latitudes and the age-model score was high, we had no reason to exclude such records from the final compilation. Two high-latitude CMAR records from ODP Sites 1208 (North Pacific) and 1171 (South Pacific)^18^, scored 2 in our criteria-based scoring system, with only one nannofossil tie point between 3–5 Ma. Although not included in the final data compilation, these records are still relevant to our discussions, since they provide insights into the latitudinal expansion of the biogenic bloom event and ocean circulation patterns during the early Pliocene. Records from the compilation by Si and Rosenthal (2019)^18^, mostly had one (nannofossil) biostratigraphic tie point between 3–5 Ma and therefore scored 2 in our age model criteria. The few sites that had at least two tie points for this interval, had been smoothed or had very few samples close to the interval of interest. Only ODP Site 999 scored 3, but was excluded due to its location in a restricted basin.

## Supplementary information

Supplementary Information

## Data Availability

Additional data figures and tables that are part of this study can be found in the Supplementary Information. Data generated in this study are provided with this paper and will also be made available upon publication of the manuscript at PANGAEA under accession code: 10.1594/PANGAEA.935686. Source data are provided with this paper.

## Source data

Source Data
