# Supplementary material for: Abrupt conclusion of the late Miocene-early Pliocene biogenic bloom at 4.6-4.4 Ma
Source: Nat Commun. 2022 Jan 17;13:353. doi: 10.1038/s41467-021-27784-6 (PMC8764042; doi:10.1038/s41467-021-27784-6)
Supplement: Supplementary file 1 — Supplementary Information [file 41467_2021_27784_MOESM1_ESM.pdf]

Supplementary Information

Abrupt conclusion of the late Miocene-early Pliocene Biogenic Bloom at 4.6-4.4 Ma

B. – Th. Karatsolis[1]*, B.C. Lougheed[1], D. De Vleeschouwer[2,3] and J. Henderiks[1]

1 Department of Earth Sciences, Uppsala University, Uppsala, Sweden

2 MARUM-Center for Marine and Environmental Sciences and Department of Geosciences, University of Bremen, Bremen, Germany

3 Institute of Geology and Palaeontology, Westfälische Wilhelms-Universität, University of Münster, Münster, Germany

Contents of this file

Supplementary Notes 1 to 2

Supplementary Figures 1 to 9

Supplementary Tables 1 to 3

Summary

This supporting information document provides additional notes on the methodology used to compile the data and the records that were considered for this compilation (Supplementary Note 1, Supplementary Figures 1-5, Supplementary Tables 1-2), as well as on the calculation of number of days exceeding an irradiance threshold (Supplementary Note 2, Supplementary Figure 6). We also provide additional terrestrial monsoonal records and information about their age model scores, as well as other paleoclimatic proxy records and nannofossil evidence (Supplementary Figures 7-9, Supplementary Table 3).

Supplementary Note 1. Methods – standardization of PP proxy records

Before compiling multiple paleoproductivity records, each individual record was standardized by assigning a z-score for each data point. This was achieved using the formula:

$$z_i = \frac{x_i - \bar{x}}{S}$$

where x_i represents the ith data point in each record, $\bar{x}$ the record's mean and S the record's standard deviation. Thereafter, the z-scored data for each record were grouped in 40 kyr time bins. The chosen time bin falls close to the average temporal resolution (median number of samples / Myr) of all the records we used (Supplementary Table 1). Different time bins were tested, with no significant differences in the obtained result (Supplementary Figure 5).

Supplementary Note 2. **Calculation of number of days exceeding an irradiance threshold (Figure 3a)**

24-hr mean irradiance was calculated at 0.1 tropical day resolution (assuming a total of 365.2 days) using the scripts developed by Lougheed[1] (2021). These scripts use the approach of Berger[2] (1978), which calls for geocentric solar longitude (λ). The λ associated with each 0.1 tropical day interval is calculated following the standard Keplerian methods detailed by Meeus[3] (1998), using a binary search method to solve the Kepler equation developed by Sinnott[4] (1985). Subsequently, the number of 0.1 day increments exceeding a certain irradiance threshold can be identified (Supplementary Figure 6). All calculations assume a solar constant of 1361 Wm-2 and use the Laskar et al.[5] (2004) solutions for orbital parameters.

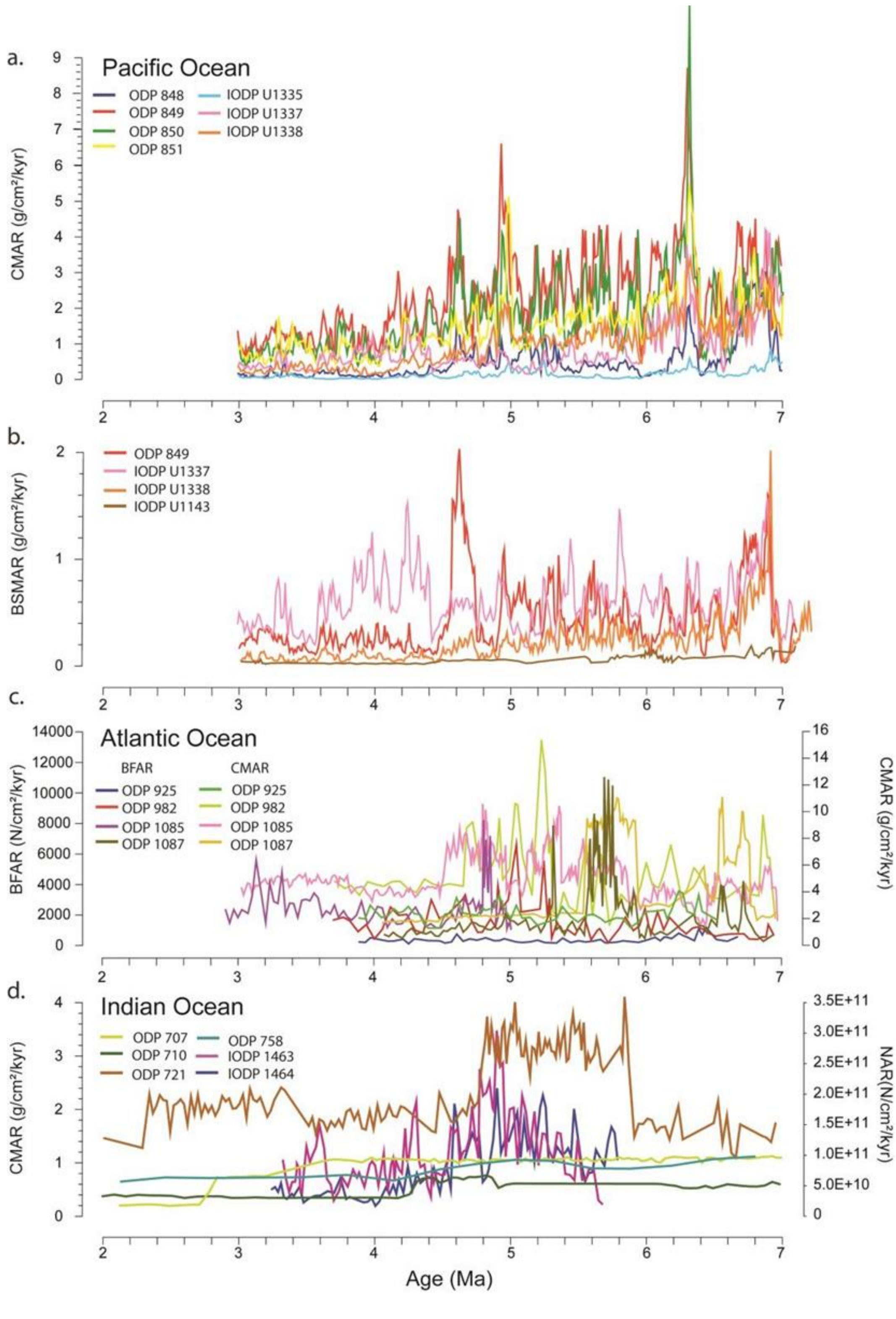

Supplementary Figure 1. Vetted (score 3-5) original (mass) accumulation rate time-series grouped by ocean basin. a. $CaCO_3$ (CMAR) and b. biogenic silica (BSMAR) mass accumulation rates from the Pacific Ocean. c. CMAR and benthic foraminifera accumulation rates (BFAR) from the Atlantic Ocean. d. CMAR and total nannoplankton accumulation rates (NAR) from the Indian Ocean. See Supplementary Tables 1-2 for details on individual records.

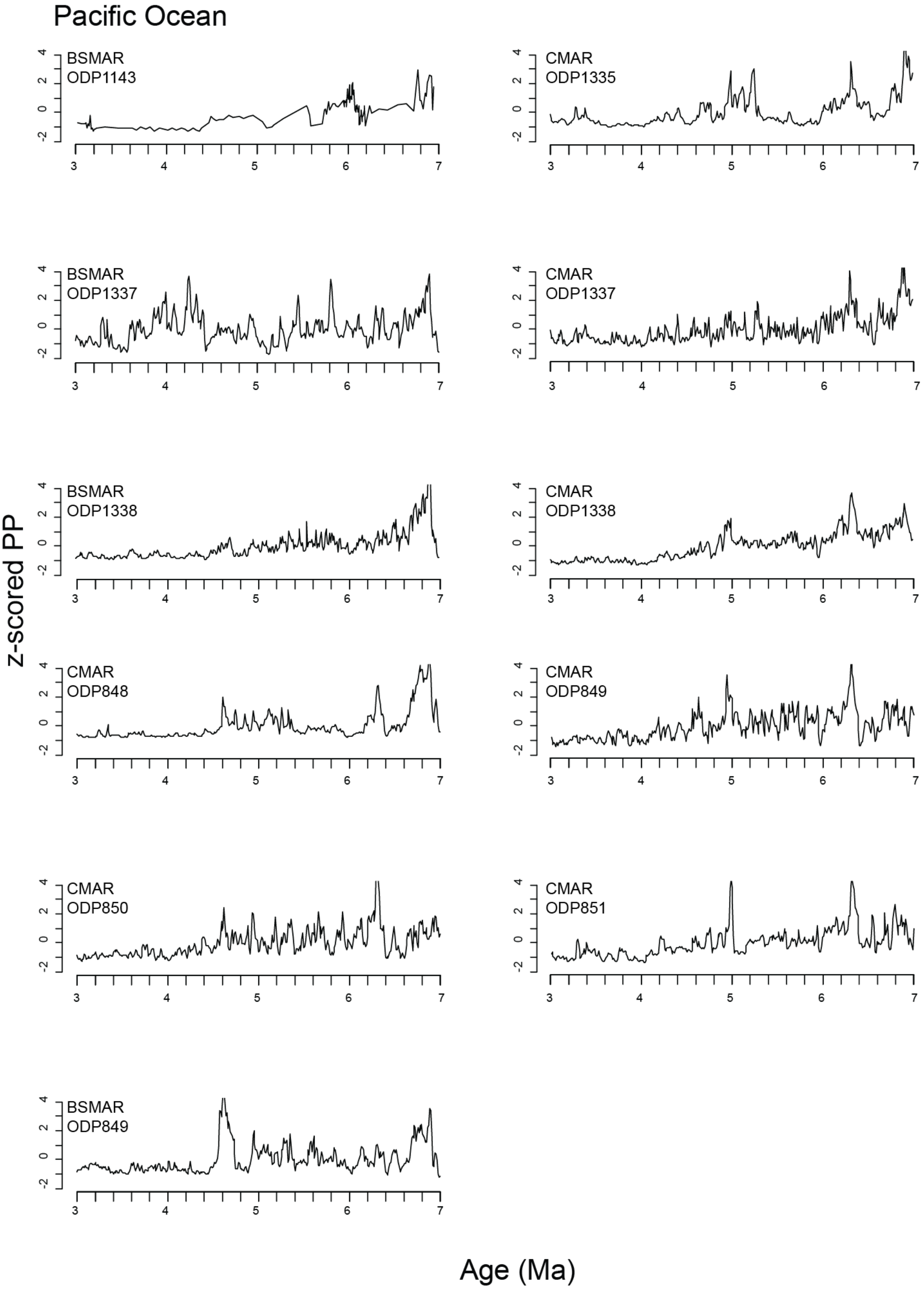

Supplementary Figure 2. Individual standardized marine paleoproductivity (PP) records for the Pacific Ocean. BSMAR= Biogenic Silica Mass Accumulation Rates, CMAR= $CaCO_3$ Mass Accumulation Rates.

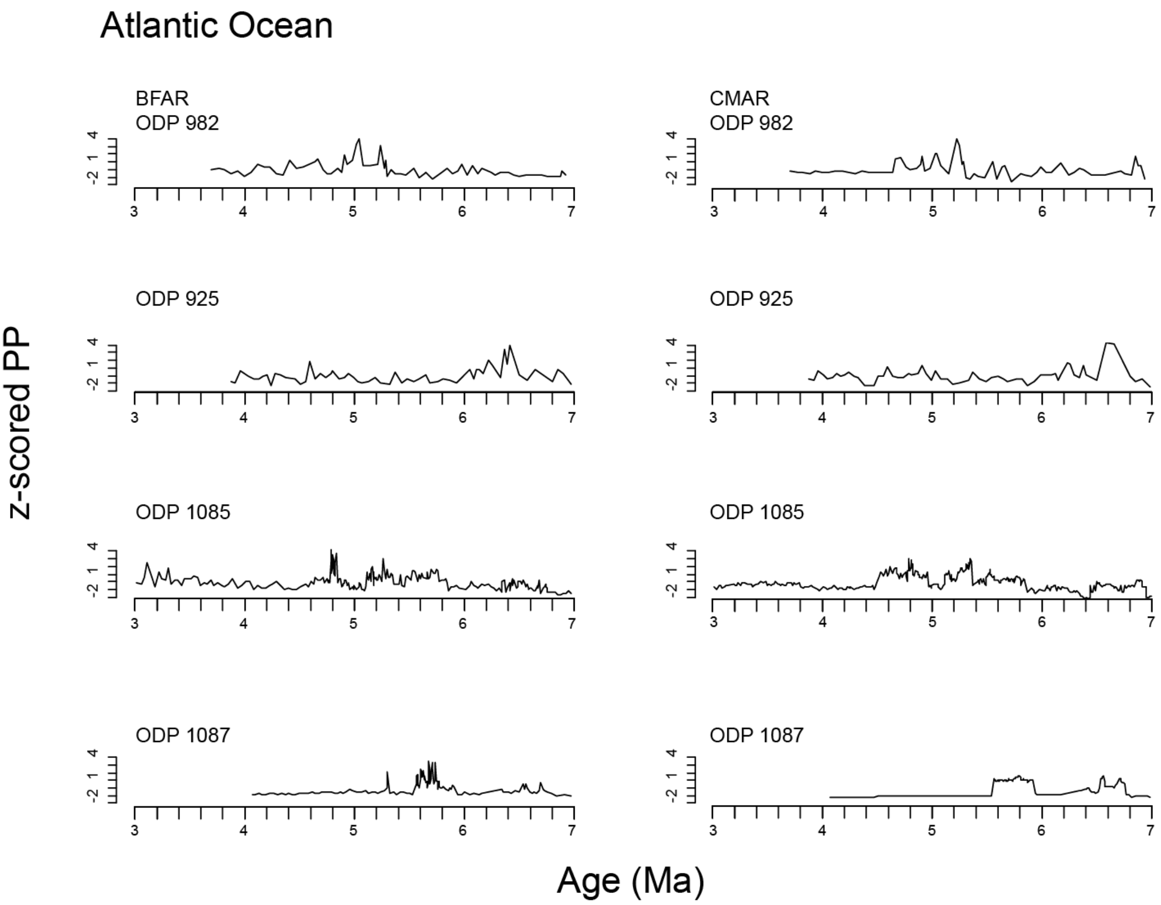

Supplementary Figure 3. Individual standardized marine paleoproductivity (PP) records for the Atlantic Ocean. BFAR= Benthic Foraminifera Accumulation Rates, CMAR= $CaCO_3$ Mass Accumulation Rates.

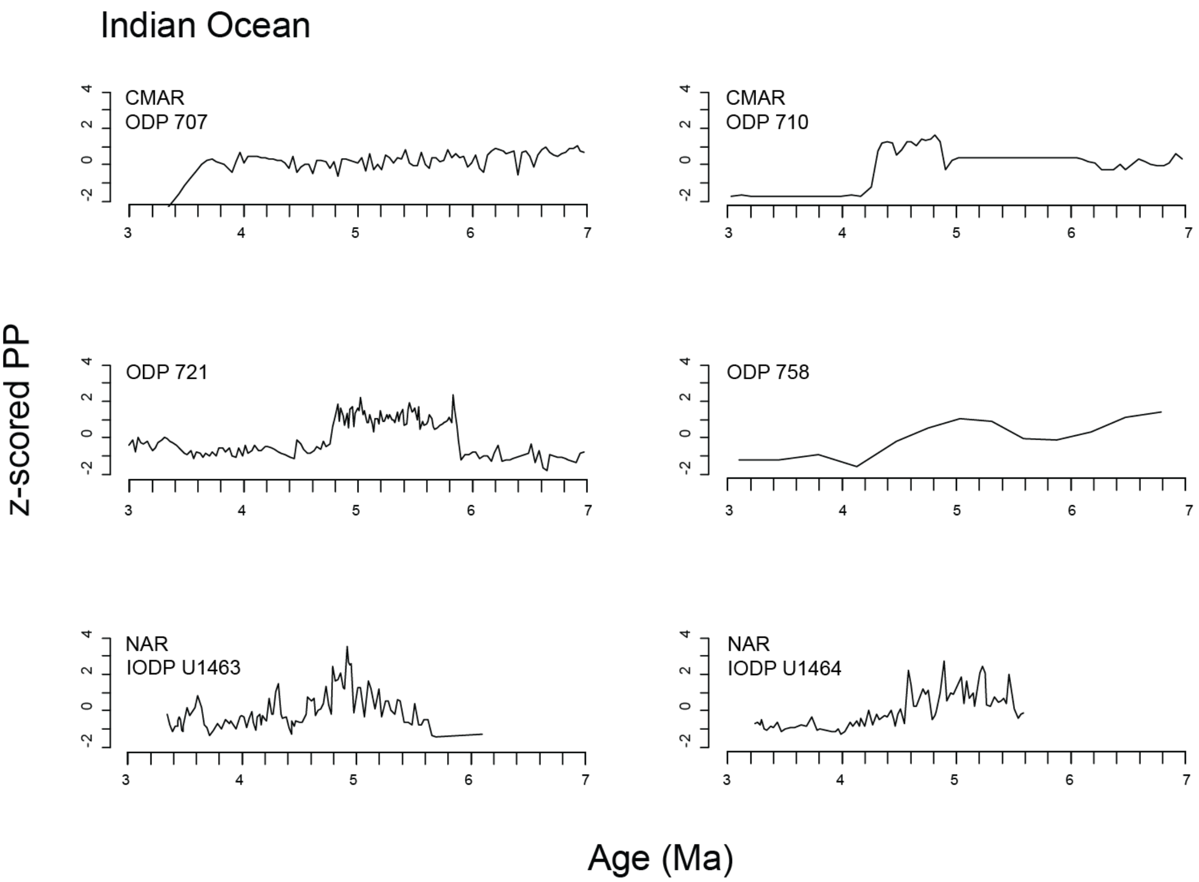

Supplementary Figure 4. Individual standardized marine paleoproductivity (PP) records for the Indian Ocean. NAR= Nannofossil Accumulation Rates, CMAR= $CaCO_3$ Mass Accumulation Rates.

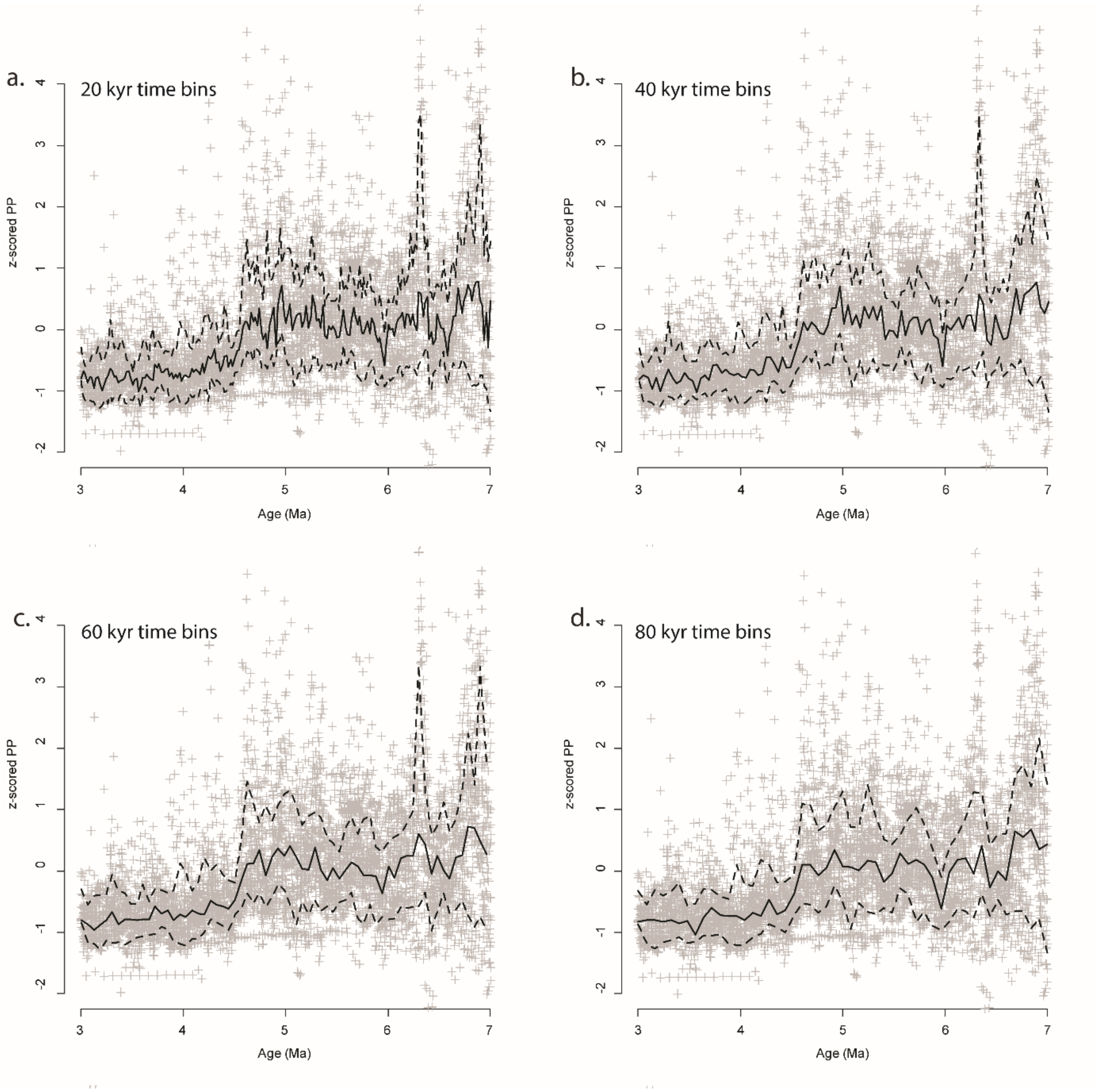

Supplementary Figure 5. Median standardized marine paleoproductivity (PP) compilation record from all ocean basins, using different time bins of: a.20 kyr; b. 40 kyr (as presented in main text); c. 60 kyr; d. 80 kyr. The choice of time bin does not affect the main findings.

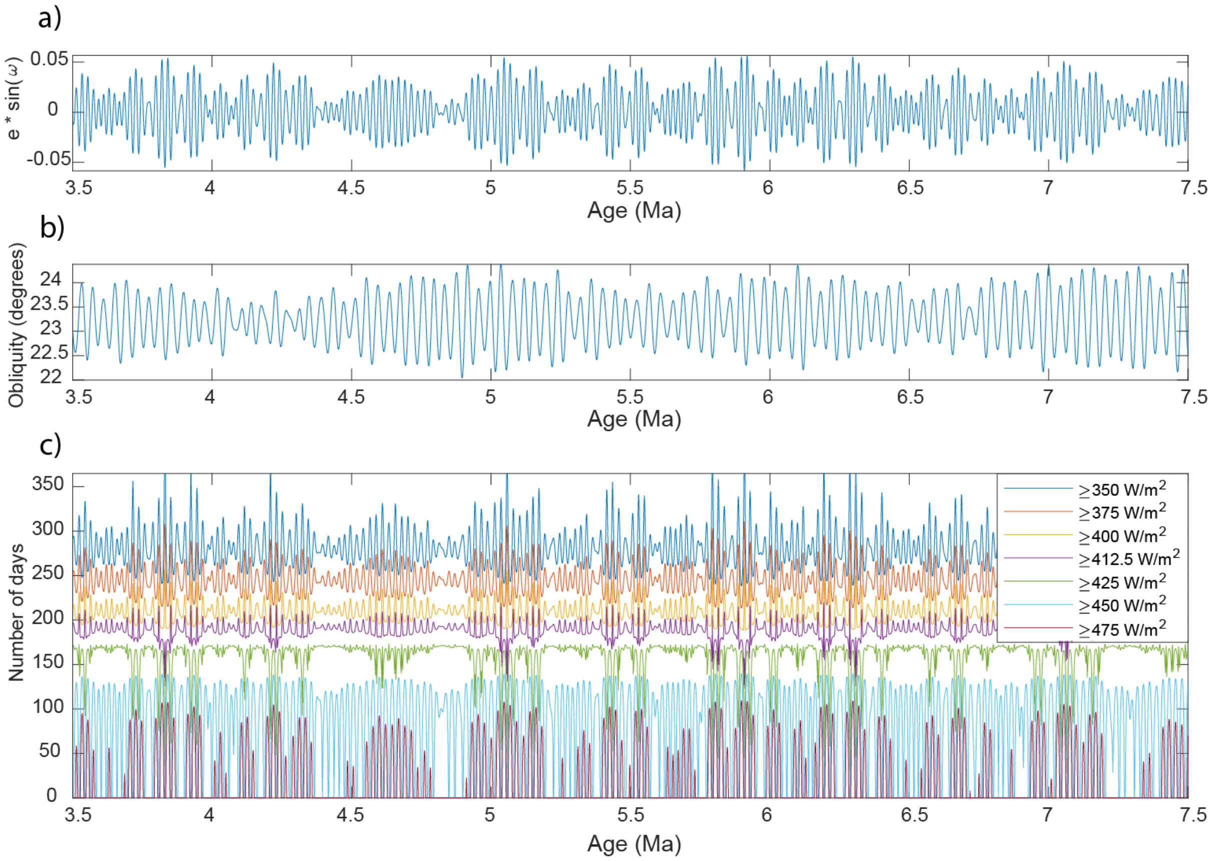

Supplementary Figure 6. Earth's rotation parameters for the late Miocene to early Pliocene interval (7.5-3.5 Ma) a. Earth's eccentricity modulated precession. b. Earth's obliquity (axial tilt) in degrees c. Number of days with irradiance higher than a certain value (W/m^2).

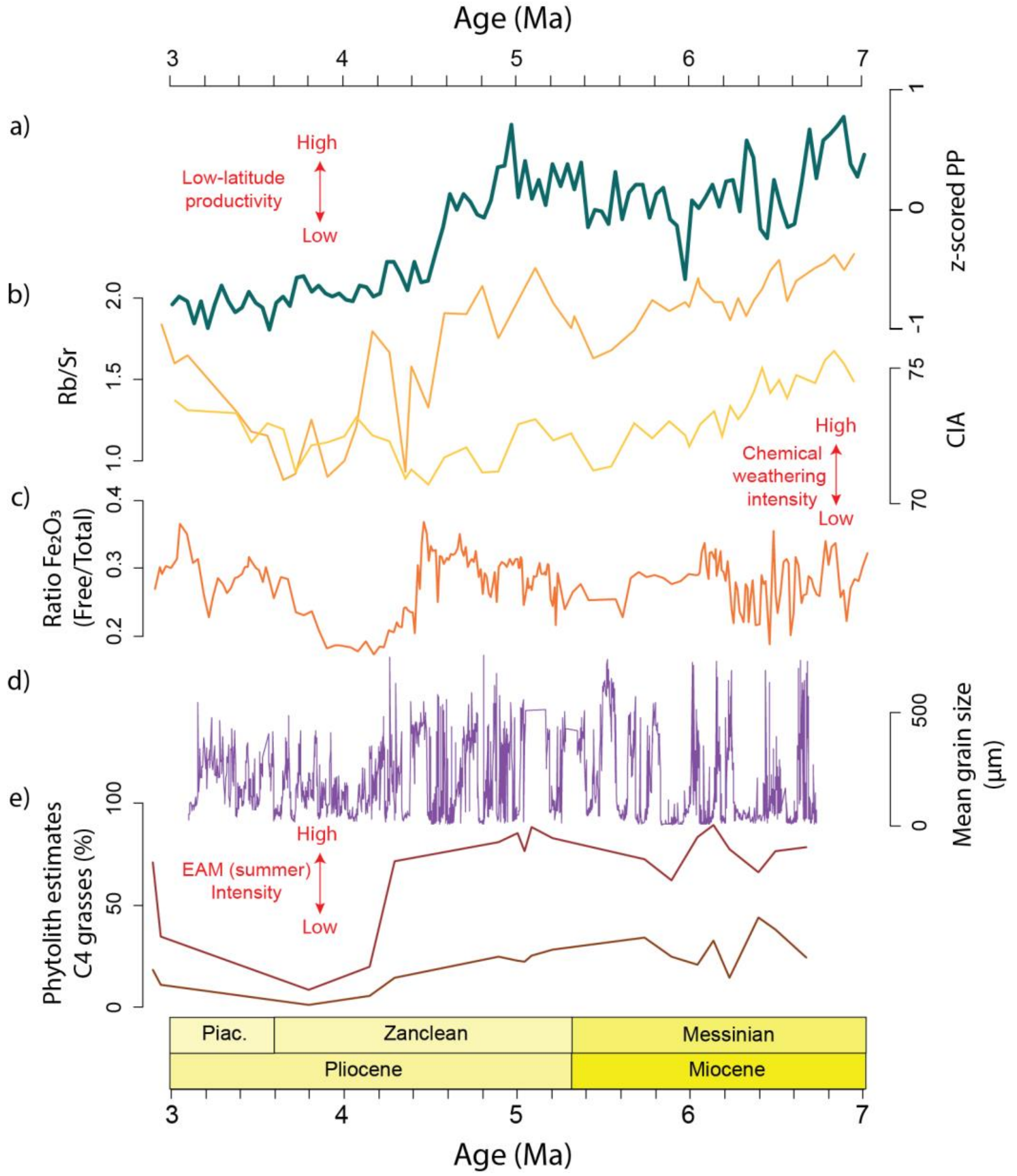

Supplementary Figure 7. Comparison of marine paleoproductivity with terrestrial and marine records of monsoonal intensity. a. Standardized median PP as compiled in this study (green line; compare Figure 3). b. Rb/Sr ratio (orange) and the CIA index (yellow), as recorded at South China Sea ODP Site 1146. Both proxies are used to describe variations in EAM intensity[6]; c. Ratio of free-to-total iron (Fe2O3) content of the Pianguan 'red clay' sequence, used as a proxy for chemical weathering intensity and EAM strength[7]; d. Mean grain sizes (µm) of the Changgoucun sedimentary sequence in central China, used to indicate precipitation and EASM intensity[8]; e. Minimum and maximum phytolith estimates of C4 grasses (%) from Weihe basin in central China, used as indicators of precipitation intensity[9].

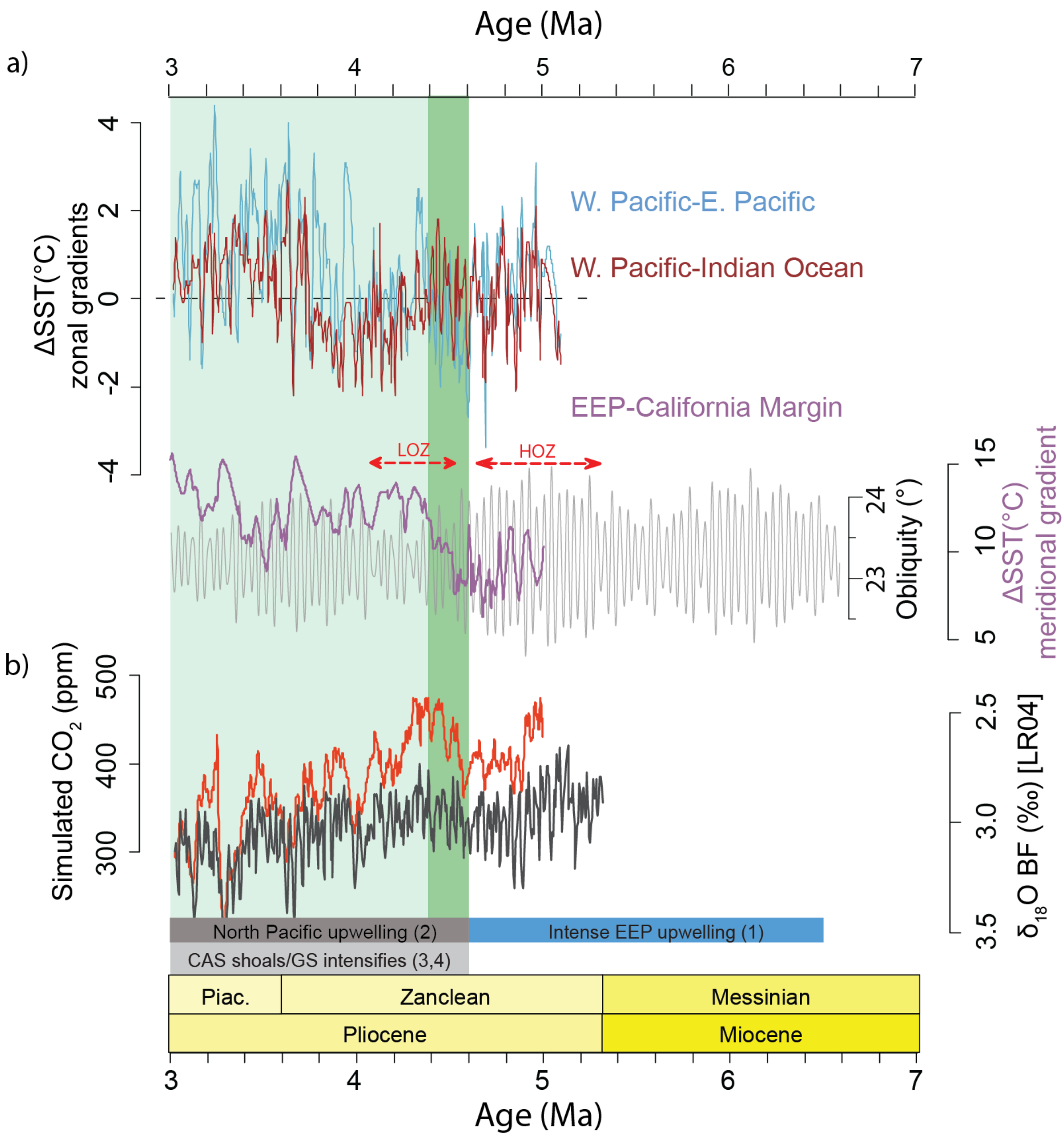

Supplementary Figure 8. Early Pliocene paleoclimate proxy records. a. Zonal SST gradients (ΔSST, °C) between ODP Site 806 (West Pacific) and ODP Site 847 (East Pacific)[10,11] (blue line) and between ODP Site 806 (West Pacific) and ODP Site 214 (Indian Ocean)[10–12] (red line). Below, the SST gradient between ODP Site 847 (EEP) and ODP Site 1021 (California Margin)[10,13], (purple line) superimposed on the Laskar 2004 obliquity solution[5] (grey line). b. The benthic foraminifera $\delta^{18}O$ stack (black, [LR04][14]) and an early Pliocene atmospheric CO_2 simulation[15] (red line). Colored horizontal bars indicate: (1) Period of intensified EEP upwelling[16], (2) Period of North Pacific upwelling intensification[17]. (3,4) Main phases of Central American Seaway (CAS) shoaling and Gulf Stream (GS) intensification[18,19]. Vertical green shadings highlight the early Pliocene PP decline (4.6-4.4 Ma) and sustained lower PP in its aftermath as identified in this study.

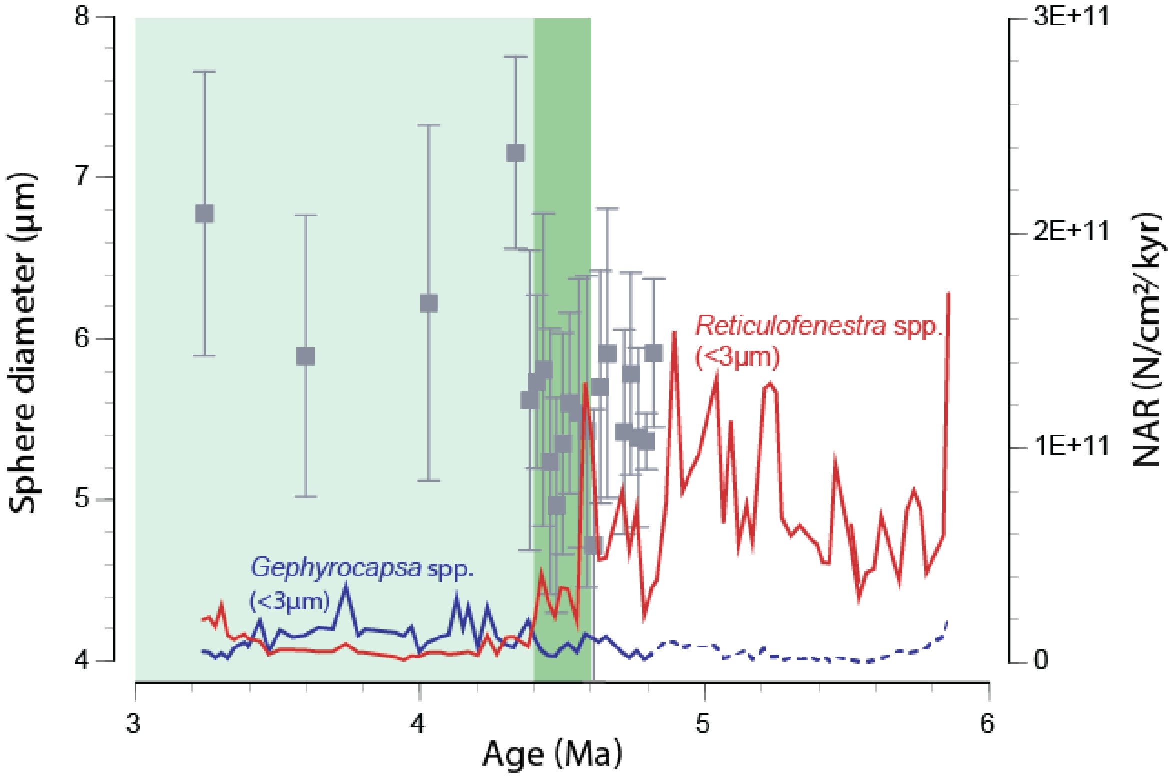

Supplementary Figure 9. Coccolith burial fluxes and mean coccosphere diameter of the most dominant taxa at IODP Site U1464[20,21] (NW Australian shelf). NAR of small (<3 µm) *Reticulofenestra* species (red line) and small (<3 µm) *Gephyrocapsa* species (blue line). The first common occurrence (FCO) of the latter occurs during the early Pliocene, and dashed line indicates time of uncertain or only very low presence of this taxon. Mean coccosphere diameter (µm; grey squares) was calculated in 21 samples across the interval of interest[21]. Error bars represent ± 1 s.d.

Supplementary Table 1. List of ODP and IODP sites and proxy data records retained in the compilations presented in this study. Source reference for age model, median sample resolution (in samples/ Myr) and the assigned score (Table 1) are given for each record from the Pacific (1-11), Atlantic (12-19) and Indian Ocean (20-25). CMAR = carbonate mass accumulation rate; BSMAR = biogenic silica mass accumulation rate; BFAR = benthic foraminifer accumulation rate; NAR = nannofossil accumulation rate.

Number	Leg-Site	Data type	Age model reference	Samples/ Myr	Score (Table 1)
1	138-848	CMAR	Lyle et al., 2019[22]	100	5
2	138-849	CMAR	Lyle et al., 2019	100	5
3	138-849	BSMAR	Lyle et al., 2019	100	5
4	138-850	CMAR	Lyle et al., 2019	100	5
5	138-851	CMAR	Lyle et al., 2019	100	5
6	320/321-U1335	CMAR	Lyle et al., 2019	100	5
7	320/321-U1337	CMAR	Lyle et al., 2019	100	5
8	320/321-U1337	BSMAR	Lyle et al., 2019	100	5
9	320/321-U1338	CMAR	Lyle et al., 2019	100	5
10	320/321-U1338	BSMAR	Lyle et al., 2019	100	5
11	184-1143	CMAR	Wang et al., 2004	33	4
12	177-982	CMAR	Diester Haass et al., 2005[23]	21	4
13	177-982	BFAR	Diester Haass et al., 2005	21	4
14	177-925	CMAR	Diester Haass et al., 2005	20	4
15	177-925	BFAR	Diester Haass et al., 2005	20	4
16	177-1085	CMAR	Diester Haass et al., 2004	92	5
17	177-1085	BFAR	Diester Haass et al., 2004	60	5

18	177-1087	CMAR	Diester Haass et al., 2004	54	4
19	177-1087	BFAR	Diester Haass et al., 2004	44	4
20	115-707	CMAR	Dickens and Owen 1999[24]	27	3
21	115-710	CMAR	Dickens and Owen 1999	17	3
22	117-721	CMAR	Dickens and Owen 1999	42	3
23	121-758	CMAR	Dickens and Owen 1999	4	3
24	356-1463	NAR	Groeneveld et al., 2021[25]	37	4
25	356-1464	NAR	Karatsolis et al., 2020[20]	33	4
				Average=~52 kyr	

Supplementary Table 2. List of DSDP, ODP and IODP records that were evaluated, but not included in the data compilation. Source reference for age model and the assigned score (Table 1) are given for each record from the Pacific (26-38), Atlantic (39-51) and Indian Ocean (51-58). CMAR = carbonate mass accumulation rate; BSMAR = biogenic silica mass accumulation rate; PMAR = phosphorus mass accumulation rate.

Number	Leg-Site	Data type	Age model reference	Score (Table 1)
26	130-803	CMAR	Si and Rosenthal 2019[26]	2
27	130-804	CMAR	Si and Rosenthal 2019	2
28	130-806	BSMAR	Si and Rosenthal 2019	2
29	130-807	CMAR	Si and Rosenthal 2019	2
30	90-590	CMAR	Si and Rosenthal 2019	2
31	90-593	CMAR	Si and Rosenthal 2019	2
32	189-1171	CMAR	Lyle et al., 2019	2
33	198-1208	CMAR	Lyle et al., 2019	2
34	115-588	CMAR	Si and Rosenthal 2019	2
35	138-846	CMAR	Si and Rosenthal 2019	2
36	138-847	CMAR	Si and Rosenthal 2019	2

37	138-852	CMAR	Si and Rosenthal 2019	2
38	138-853	CMAR	Si and Rosenthal 2019	2
39	177-1086	CMAR	Diester Haass et al., 2004[27]	2
40	94-607	CMAR	Si and Rosenthal 2019	2
41	94-608	CMAR	Si and Rosenthal 2019	2
42	149-558	CMAR	Si and Rosenthal 2019	2
43	108-667	CMAR	Si and Rosenthal 2019	2
44	154-928	CMAR	Si and Rosenthal 2019	2
45	208-1264	CMAR	Si and Rosenthal 2019	2
46	208-1266	CMAR	Si and Rosenthal 2019	2
47	177-1088	CMAR	Si and Rosenthal 2019	2
48	72-516	PMAR	Hermoyian and Owen 2001[28]	2
49	73-522	PMAR	Hermoyian and Owen 2001	2
50	74-526	PMAR	Hermoyian and Owen 2001	2
51	165-999	CMAR	Si and Rosenthal 2019	3 (see methods)

52	115-709	CMAR	Dickens and Owen 1999	2
53	115-711	CMAR	Dickens and Owen 1999	2
54	117-722	CMAR	Dickens and Owen 1999	2
55	117-728	CMAR	Dickens and Owen 1999	2
56	121-754	CMAR	Dickens and Owen 1999	2
57	121-756	CMAR	Si and Rosenthal 2019	2
58	121-757	CMAR	Dickens and Owen 1999	2

Supplementary Table 3. List of monsoonal proxies mentioned in this study, and their assigned scores based on our age model criteria (Table 1).

Number	Location	Data type	Age model reference	Score (Table 1)
1	Central China	Phytoliths C4 (%)	An et al., 2000[29]	2
2	Central China	Mean grain size (μm)	Wang et al., 2020[8]	3
3	South China Sea	Monsoon intensity/chemical weathering proxies	Wang et al., 2000[30]	2
4	Chinese Loess Plateau	Free/Total Fe_2O_3	Yang et al., 2018[7]	3

Supplementary References

1. Lougheed, B. Orbital, the Box-An interactive educational tool for in-depth understanding of astronomical climate forcing. *EarthArxiv* (2021).
2. Berger, A. L. Long-Term Variations of Daily Insolation and Quaternary Climatic Changes. *J. Atmos. Sci.* **35**, 2362–2367 (1978).
3. Meeus, J. *Astronomical Algorithms*. (Willman-Bell, 1998).
4. Sinnott, R. W. A computer assault on Kepler's equation. *Sky Telescope* **70**, 158–159 (1985).
5. Laskar, J. *et al.* A long-term numerical solution for the insolation quantities of the Earth. *Astron. Astrophys.* **428**, 261–285 (2004).
6. Wan, S., Clift, P. D., Li, A., Li, T. & Yin, X. Geochemical records in the South China Sea: Implications for East Asian summer monsoon evolution over the last 20 Ma. *Geol. Soc. Spec. Publ.* **342**, 245–263 (2010).
7. Yang, S. *et al.* A strengthened East Asian Summer Monsoon during Pliocene warmth: Evidence from 'red clay' sediments at Pianguan, northern China. *J. Asian Earth Sci.* **155**, 124–133 (2018).
8. Wang, Y. *et al.* Combined high- and low-latitude forcing of East Asian monsoon precipitation variability in the Pliocene warm period. *Sci. Adv.* **6**, 1–10 (2020).
9. Wang, H. *et al.* Asian monsoon rainfall variation during the Pliocene forced by global temperature change. *Nat. Commun.* **10**, 4–11 (2019).
10. Lariviere, J. P. *et al.* Late Miocene decoupling of oceanic warmth and atmospheric carbon dioxide forcing. *Nature* **486**, 97–100 (2012).
11. Wara, M. W., Ravelo, A. C. & Delaney, M. L. Climate change: Permanent El Niño-like conditions during the Pliocene warm period. *Science (80-.).* **309**, 758–761 (2005).
12. Karas, C. *et al.* Mid-Pliocene climate change amplified by a switch in Indonesian subsurface throughflow. *Nat. Geosci.* **2**, 434–438 (2009).
13. Dekens, P. S., Ravelo, A. C. & McCarthy, M. D. Warm upwelling regions in the Pliocene warm period. *Paleoceanography* **22**, 1–12 (2007).
14. Lisiecki, L. E. & Raymo, M. E. A Pliocene-Pleistocene stack of 57 globally distributed benthic δ 18O records. *Paleoceanography* **20**, 1–17 (2005).
15. Stap, L. B. *et al.* CO2 over the past 5 million years: Continuous simulation and new δ11B-based proxy data. *Earth Planet. Sci. Lett.* **439**, 1–10 (2016).
16. Zhang, Y. G., Pagani, M., Henderiks, J. & Ren, H. A long history of equatorial deep-water upwelling in the Pacific Ocean. *Earth Planet. Sci. Lett.* **467**, 1–9 (2017).
17. Haug, G. H., Sigman, D. M., Tiedemann, R., Pedersen, T. F. & Sarnthein, M. Onset of permanent stratification in the subarctic Pacific Ocean. *Nature* **401**, 779–782 (1999).
18. O'Dea, A. *et al.* Formation of the Isthmus of Panama. *Sci. Adv.* **2**, 1–11 (2016).
19. Auderset, A. *et al.* Gulf Stream intensification after the early Pliocene shoaling of the Central American Seaway. *Earth Planet. Sci. Lett.* **520**, 268–278 (2019).

20. Karatsolis, B. T., De Vleeschouwer, D., Groeneveld, J., Christensen, B. & Henderiks, J. The Late Miocene to Early Pliocene "Humid Interval" on the NW Australian Shelf: Disentangling climate forcing from regional basin evolution. *Paleoceanogr. Paleoclimatology* **35**, (2020).

21. Eliassen, N. Cell Size Variation in Fossil Coccolithophores (Haptophyta): A Study of Pliocene Sediments from Northwestern Australia. (Uppsala University, 2018).

22. Lyle, M., Drury, A. J., Tian, J., Wilkens, R. & Westerhold, T. Late Miocene to Recent High Resolution Eastern Equatorial Pacific Carbonate Records: Stratigraphy linked by dissolution and paleoproductivity. *Clim. Past Discuss.* 1–41 (2018) doi:10.5194/cp-2018-157.

23. Diester-Haass, L., Billups, K. & Emeis, K. C. In search of the late Miocene-early Pliocene 'biogenic bloom' in the Atlantic Ocean (Ocean Drilling Program Sites 982, 925, and 1088). *Paleoceanography* **20**, 1–13 (2005).

24. Dickens, G. R. & Owen, R. M. The Latest Miocene-Early Pliocene biogenic bloom: A revised Indian Ocean perspective. *Mar. Geol.* **161**, 75–91 (1999).

25. Groeneveld, J., De Vleeschouwer, D., McCaffrey, J. C. & Gallagher, S. J. Dating the Northwest Shelf of Australia since the Pliocene. *Geochemistry, Geophys. Geosystems* **22**, 1–20 (2021).

26. Si, W. & Rosenthal, Y. Reduced continental weathering and marine calcification linked to late Neogene decline in atmospheric CO2. *Nat. Geosci.* **12**, 833–838 (2019).

27. Diester-Haass, L., Meyers, P. A. & Bickert, T. Carbonate crash and biogenic bloom in the late Miocene: Evidence from ODP Sites 1085, 1086, and 1087 in the Cape Basin, southeast Atlantic Ocean. *Paleoceanography* **19**, 1–19 (2004).

28. Hermoyian, C. S. & Owen, R. M. Late Miocene-early Pliocene biogenic bloom: Evidence from low-productivity regions of the Indian and Atlantic oceans. *Paleoceanography* **16**, 95–100 (2001).

29. An, Z. *et al.* Red Clay sequences in Chinese loess plateau and recorded paleoclimate events of the late tertiary. *Quat. Sci.* **05**, 435–436 (2000).

30. Wang, P. X., Prell, W. L. & Al., E. Proceedings of the Ocean Drilling Program, Initial Reports. **184**, (2000).
